# Novel biomarkers used for early diagnosis and tyrosine kinase inhibitors as targeted therapies in colorectal cancer

**DOI:** 10.3389/fphar.2023.1189799

**Published:** 2023-09-01

**Authors:** Huafeng Jiang, Senjun Zhou, Gang Li

**Affiliations:** Department of Anus and Colorectal Surgery, Shaoxing People’s Hospital, Shaoxing, China

**Keywords:** colorectal cancer, diagnosis, tyrosine kinase inhibitors, biomarker, therapies

## Abstract

Colorectal cancer (CRC) is the third most common and second most lethal type of cancer worldwide, presenting major health risks as well as economic costs to both people and society. CRC survival chances are significantly higher if the cancer is diagnosed and treated early. With the development of molecular biology, numerous initiatives have been undertaken to identify novel biomarkers for the early diagnosis of CRC. Pathological disorders can be diagnosed at a lower cost with the help of biomarkers, which can be detected in stool, blood, and tissue samples. Several lines of evidence suggest that the gut microbiota could be used as a biomarker for CRC screening and treatment. CRC treatment choices include surgical resection, chemotherapy, immunotherapy, gene therapy, and combination therapies. Targeted therapies are a relatively new and promising modality of treatment that has been shown to increase patients’ overall survival (OS) rates and can inhibit cancer cell development. Several small-molecule tyrosine kinase inhibitors (TKIs) are being investigated as potential treatments due to our increasing awareness of CRC’s molecular causes and oncogenic signaling. These compounds may inhibit critical enzymes in controlling signaling pathways, which are crucial for CRC cells’ development, differentiation, proliferation, and survival. On the other hand, only one of the approximately 42 TKIs that demonstrated anti-tumor effects in pre-clinical studies has been licensed for clinical usage in CRC. A significant knowledge gap exists when bringing these tailored medicines into the clinic. As a result, the emphasis of this review is placed on recently discovered biomarkers for early diagnosis as well as tyrosine kinase inhibitors as possible therapy options for CRC.

## 1 Introduction

Colorectal cancer (CRC) is a third primary global health concern and a second leading cause of cancer-related deaths worldwide, which poses financial burdens on people and society (Morgan et al., 2023). In all clinical practice and research disciplines, CRC, including rectal and colon cancer, is treated as a single tumor type (Alzahrani et al., 2021). CRC originates from the lining of the colon or rectum and follows a specific pathological progression. In most cases, it typically begins as small growths called polyps, which can be detected during a colonoscopy. Over time, these polyps can develop into cancerous tumors, invading the surrounding tissues and potentially spreading to distant organs, frequently the liver. Over 10–15 years, this process necessitates the accumulation of genetic mutations that can be somatic or germ-line in nature (Lotfollahzadeh et al., 2022). Some common risk factors for CRC include family, genetic, geriatric, nutritional, lifestyle, and environmental variables. Inflammatory bowel conditions, including Crohn’s disease and ulcerative colitis, are additional risk factors. Moreover, issues with inactivity, obesity, smoking, and alcohol usage can be resolved (Sawicki et al., 2021).

### 1.1 Incidence and mortality

Certain pathological features, such as adenomatous polyps or advanced stages of carcinoma, are associated with an increased disease prevalence (Akimoto et al., 2021). The incidence and mortality of CRC differ significantly by country and region worldwide. According to the data from Global Cancer Observatory (GLOBOCAN), there were around 1.9 million new cases of CRC and 930,000 fatalities in 2020 worldwide. The incidence rates were lowest in several African countries and Southern Asia, while the highest incidence rates were reported from Europe, Australia, and New Zealand regions (Morgan et al., 2023). Similar trends were observed in CRC mortality rates, with Southern Asia having the lowest rates (2.5 per 100,000 females) and Eastern Europe having the highest rates (20.2 per 100,000 males). Additionally, there was a 10-fold difference in incidence rates between males and females in all countries. Males showed higher incidence and fatality rates than females (Wong et al., 2021). By 2040, CRC is expected to cause 3.2 million new cases and 1.6 million fatalities, mostly in high-HDI (human development index) countries (Xi and Xu., 2021). The incidence and mortality of CRC have decreased, and the US now ranks among the third-highest HDI countries. In the US, stage I colon cancer has a 5-year relative survival rate of around 92%. Stage IIA and IIB exhibit rates of 87% and 65%, respectively. Surprisingly, stage IIIA and stage IIIB have slightly greater 5-year survival rates, at 90% and 72%, respectively. While stage IV, or metastatic CRC (mCRC), has a 5-year survival rate of only 12%, stage IIIC has a survival rate of 53%. With 88% for stage I, 81% for stage IIA, 50% for stage IIB, 83% for stage IIIA, 72% for stage IIIB, 58% for stage IIIC, and 13% for stage IV, the 5-year survival rates for rectal cancer seem to be slightly lower. These stages are based on the TNM system’s previous version. The unexpected increase in survival from stage II to stage III tumors can be attributed to the technique utilized to diagnose and treat different types of CRC (Rawla et al., 2019).

A higher prevalence of the disease underscores the need for effective screening programs and public awareness campaigns to promote early detection, as it significantly improves the prognosis and treatment outcomes for individuals with CRC (Stark et al., 2020). Over the past few decades, there has been remarkable progress in understanding the molecular basis of CRC, leading to the identification of novel biomarkers for early diagnosis and the development of targeted therapies, such as tyrosine kinase inhibitors (TKIs), for the treatment of CRC (Bresalier et al., 2020). Early diagnosis is critical in improving patient outcomes by enabling timely intervention and reducing mortality rates. Traditional screening methods for CRC, such as colonoscopy and fecal occult blood tests, have effectively detected early-stage tumors and precancerous lesions (Huang and Yang., 2022). However, these methods often have invasiveness, cost, and patient compliance limitations. Therefore, there is an urgent need to explore and validate novel biomarkers that can enhance the sensitivity and specificity of CRC detection while offering non-invasive and cost-effective alternatives (Shaukat and Levin., 2022).

### 1.2 Role of biomarkers in CRC

Biomarkers have been developed to aid in identifying patient responses to cancer diagnosis, therapy, and monitoring (Oh and Joo., 2022). Biomarkers, which include genetic alterations, epigenetic modifications, and protein expression patterns, hold immense promise as tools for CRC screening, risk assessment, and prognosis prediction. These biomarkers can be detected not only in solid tissue samples but also in blood and/or stool, allowing for non-invasive and convenient testing (Shen et al., 2022). The development of high-throughput genomic technologies has revolutionized biomarker discovery and enabled the identification of candidate markers associated with CRC initiation, progression, and response to treatment. Furthermore, integrating multiple biomarkers into diagnostic algorithms can improve the accuracy and reliability of CRC screening, facilitating the implementation of personalized medicine approaches (Islam Khan et al., 2022). Moreover, liquid biopsy approaches, which involve the analysis of circulating tumor DNA (ctDNA) and microRNAs (miRNAs), have gained significant attention as non-invasive methods for CRC screening and monitoring (Zhou et al., 2022).

### 1.3 Diagnosis and treatment of CRC

In addition to early diagnosis, targeted therapies have revolutionized the treatment landscape for CRC patients. Emerging evidence suggests a potential correlation between CRC and the use of TKIs. Tyrosine kinases play a crucial role in cell signaling pathways, and their dysregulation has been implicated in various types of cancers, including CRC (Thomson et al., 2022). TKIs are a class of drugs that selectively inhibit the activity of specific tyrosine kinase enzymes involved in CRC pathogenesis and progression. The aberrant activation of signaling pathways, such as the epidermal growth factor receptor (EGFR) and vascular endothelial growth factor receptor (VEGFR) pathways, has been implicated in CRC tumorigenesis and angiogenesis (Iyer et al., 2022). While TKIs have shown promising results in treating certain cancers, including gastrointestinal stromal tumors, their efficacy in CRC has been more limited. Studies have indicated that specific genetic mutations and alterations in tyrosine kinase signaling pathways may influence the response to TKIs in CRC patients (Xie et al., 2020). TKIs targeting these pathways, either as monotherapy or combined with standard chemotherapy regimens, have shown clinical efficacy in various studies (Hossain et al., 2022).

This review article will provide a comprehensive overview of the recent advancements in novel biomarkers used for early diagnosis of CRC. We will explore the potential of genetic alterations, epigenetic modifications, and other molecular markers as diagnostic tools in CRC. We will discuss the application of specific genetic markers, such as mutations in the Adenomatous polyposis coli (APC), Kirsten rat sarcoma (KRAS), and tumor protein p53 (TP53) genes, as well as epigenetic including DNA methylation patterns and histone modifications as diagnostic and prognostic indicators. We hope to contribute to the ongoing efforts to improve CRC patient outcomes and facilitate precision medicine approaches by integrating the knowledge of these emerging biomarkers and therapies. Moreover, we will also provide an in-depth analysis of the current status and future perspectives of TKIs as targeted therapies in CRC treatment.

## 2 Novel biomarkers used for early diagnosis of CRC

Biomarkers are commonly used in CRC diagnostics to detect the presence of biochemical compounds that circulate in the body. These compounds may include gut microorganisms, miRNA in the blood, tumor-derived cells, tumor DNA, and proteins.

### 2.1 The gut microbiome as biomarkers

Inflammation, immunological modulation, dietary component metabolism, and exposure to genotoxic substances are the primary ways the gut microbiota contributes to cancer. Patients with CRC have a range of unique microbiomes that can be used as biomarkers for the diagnosis, prognosis, and treatment efficacy (Wong and Yu., 2019). There has been a lot of interest lately in the possible connection between gut bacteria and CRC.

#### 2.1.1 Gut fungi

Gut fungi, specifically the dysbiosis or imbalance in the fungal community, have been implicated in CRC development. Recent studies have identified specific fungal biomarkers, such as *Candida tropicalis* and Debaryomyces hansenii, whose overgrowth or altered abundance in the gut may serve as potential molecular indicators of CRC, offering insights into its pathogenesis and possible diagnostic strategies. The overgrowth of gut fungi in CRC patients can be weakened immune system function and change the microenvironment of the colon, creating an environment conducive to fungal proliferation and colonization (Plaza-Díaz et al., 2021). However, there is insufficient information on the fungus microbiome in CRC. The top 3 fungi highly enriched in CRC were Phanerochaete chrysosporium, Lachancea waltii, and Aspergillus rambellii. It has been established that fungi anomalies in feces are associated with CRC (Gao et al., 2022) and in healthy individuals compared to CRC patients. It was observed that the proportion of Basidiomycota or Ascomycota was higher in CRC patients than in healthy individuals.

Moreover, two fungi, Pneumocystis and *Saccharomyces cerevisiae*, which have beneficial effects on the gut and possess anti-inflammatory properties, were found to be reduced in CRC patients. Conversely, Malasseziomycetes (fungi) were more abundant in healthy individuals than in CRC patients (Liu et al., 2022). Furthermore, the researchers noted that patients exhibited notably elevated levels of *Candida* albicans, a type of yeast. According to Stary et al. (2020), individuals who are at risk for CRC or have early asymptomatic with CRC may find it helpful to use C. albicans yeast as a diagnostic marker (Starý et al., 2020).

#### 2.1.2 Gut bacteria

Gut bacteria, particularly the alterations in the composition and diversity of the bacterial community, have been linked to CRC development. The molecular mechanisms underlying this association involve the production of specific metabolites, such as short-chain fatty acids and secondary bile acids, as well as the activation of pro-inflammatory pathways, which can serve as potential biomarkers for the detection and monitoring of CRC (Xie et al., 2020). In a large-scale study, researchers discovered that individuals with CRC had an increased population of certain bacteria, including *Fusobacterium* nucleatum, Porphyromonas asaccharolytica, *Bacteroides fragilis*, Parvimonas micra, Prevotella intermedia, Alistipes finegoldii, and Thermanaerovibrio acidaminovorans. These seven bacteria are potential markers for diagnosing CRC (Chang et al., 2021). Adenomas were found to have unusually elevated levels of the “m3” product, particularly those originating from *Clostridium* hathewayi (Ch), *Fusobacterium* nucleatum (Fn), and Lachnoclostridium. Only these three bacteria have been identified in feces as markers for colorectal adenomas and cancers (Chan and Liang., 2022). Actinomyces odontolyticus and Atopobium parvulum were exclusively found in polypoid adenomas and/or intramucosal carcinomas (early stage), indicating the wide availability of Fn enhanced slowly from intramucosal carcinoma to early CRC (Liu et al., 2022). This discovery raises the possibility of employing these microorganisms as stool-screening indicators.

#### 2.1.3 Gut viruses

The role of gut viruses in CRC is still being explored. Emerging evidence suggests that certain viral infections, such as high-risk human papillomavirus (HPV) and Epstein-Barr virus (EBV), may contribute to the development and progression of CRC through various molecular mechanisms, including viral integration into the host genome, dysregulation of host cell signaling pathways, and evasion of immune surveillance, providing potential avenues for viral-based biomarker identification in CRC diagnosis and treatment (Koonin et al., 2021). Most cancer-associated bacteriophages were temperate, demonstrating a connection between bacteriophage communities and CRC and the possibility that they could influence cancer progression by altering bacterial host populations (Hannigan et al., 2018). A similar study confirmed the association between viral indicators and CRC by observing a substantial rise in the variety of gut bacteriophage populations in feces from CRC patients and controls. Numerous studies have shown how closely microbes and cancer are related and how gut bacteria have opened up new possibilities for CRC detection (Handley and Devkota., 2019). However, a common microbiome biomarker has not been used to diagnose CRC because there is not yet a universally accepted standard for discovering microbiota. In order to enhance CRC diagnosis in the future, investigators must examine multiple microbiomes in patients from various ethnic groups since a microbe might not be capable of predicting CRC with adequate precision (Ding et al., 2022).

### 2.2 Volatile organic compounds (VOCs) as biomarkers

The ability to distinguish between diseases based on their “smell” has gained popularity as a research topic in recent years due to the growing interest in the “smell” of diseases. A potential early CRC screening method involves the detection of VOCs, which are non-invasive biomarkers. Multiple investigations have revealed several VOCs as CRC biomarkers (Ding et al., 2022). Furthermore, changes in gut flora have a direct impact on the profile of VOC generation.

#### 2.2.1 Fecal VOCs

Propan-2-ol, produced from ethyl 3-methyl-butanoate, hexane-2-one, and acetone, produced when ethanol and 3-methylbutaninoic react, positively correlates with the diagnosis of CRC, according to a 3-fecal volatile organic compound panel (Bond et al., 2019). One stool VOC contributing to CRC formation is hydrogen sulphide (HS). Microorganisms in the gut and internal enzymatic activities in the colon generate hydrogen sulfide. Higher levels of HS (over 2.4 mmol/kg) are hazardous, but lower levels are benign. The presence of higher-than-normal quantities of HS in both the lumen and the feces can throw off the equilibrium of the microbiota. For instance, this phenomenon affects patients with CRC (Lin et al., 2023). With the help of eNose Cyranose 320, patients with CRC could be characterized from controls with 85% sensitivity and 87% specificity (AUC 0.92). Similar results were attained with selected ion flow tube mass spectrometry (SIFT-MS), which separated CRC and advanced adenoma patients from healthy controls with 75% accuracy (72% sensitivity and 78% specificity). Recently, eNose Scent A1 was even more successful in many patients (Vernia et al., 2021).

#### 2.2.2 Breath-exhaled VOCs

Using a pattern of 15 VOCs identified with gas-chromatography mass spectrometry (GC-MS), Altomare et al. differentiated between CRC patients and healthy controls with an accuracy of more than 80% (Altomare et al., 2015). A study isolated 4 volatile organic compounds: methyl octane, ethanol, ethyl acetate, and acetone, 4-. Acetone and ethyl acetate levels were more significant in patients with CRC (94% specificity and 85% sensitivity) and an accuracy of 91% (Zhang et al., 2021). The VOC samples from CRC patients also had considerably higher levels of 3-hydroxy-2,4,4-trimethylpentyl, trans-2-dodecen-1-ol, ethylaniline, cyclooctylmethanol, 4-ethyl-1-octyn-3-ol, 2,2-dimethyl decane, Cyclohexanone, and dodecane. But much lower levels of 2-methylpropanoate and 6-t-butyl-2,2,9,9 tetramethyl-3,5-decadien-7-yne (Amal et al., 2016).

#### 2.2.3 Urinary VOCs

In a larger sample of 562 people, the diagnostic efficacy of urine VOCs detected by Field Asymmetric Ion Mobility Spectrometry (FAIMS) was less accurate than that of the fecal immunochemical test (FIT) (80% sensitivity and 93% specificity vs. 63% sensitivity and 63% specificity, respectively). One study found that CRC patients had significantly higher concentrations of 2-methyl-3-phenyl-2-propenal, 2,7-dimethyl-quinoline, and 1,4,5-trimethyl-naphthalene (Vernia et al., 2021).

### 2.3 Tissue biomarkers

#### 2.3.1 Cadherin-17 (CDH17)

CDH17 is a glycoprotein that spans the cell membrane and requires calcium to function properly. Its primary purpose is to aid tissues in preserving their typical structure under normal conditions. The immunohistochemistry marker CDH17 helps identify primary and metastatic colorectal adenocarcinomas (Tournier et al., 2023). According to reports, 100% of metastatic and 96%–100% of primary CRC express CDH17. CDH17 and SATB2 were excellent potential biomarkers for diagnosing metastatic colorectal adenocarcinoma and pulmonary enteric malignancy (Neri et al., 2020). The expression of CDH17 in CRC tissues and plasma gradually increased as the disease progressed to more advanced stages. There is a link between liver metastasis, high CDH17 expression, and a bad prognosis for CRC patients (Tsoi et al., 2019).

#### 2.3.2 Anti-glycoprotein 33 (GPA 33)

GPA33, a transmembrane protein overexpressed in CRC, has potential molecular mechanisms involving cell adhesion, immune evasion, and tumor growth, highlighting its significance as a biomarker and therapeutic target; however, its current clinical application as a diagnostic or prognostic tool in CRC is still under investigation and requires further validation. The tumor-associated antigen human GPA33 is expressed in approximately 95% of primary and mCRC (Markandeywar et al., 2023). It is a surface-localized, extremely persistent, and inert protein. PGA 33 has a 95.9% sensitivity and an 85.4% specificity for CRC. A33 has been the target of clinical-stage antibodies used to treat CRC (Wei et al., 2020). The new anti-A33 antibody may prevent the development of mouse CRC lung metastases, and A33-expressing murine adenocarcinoma cells may be destroyed by antibody-dependent cell-mediated cytotoxicity (ADCC) (Murer et al., 2020). The A33 had a sensitivity comparable to Caudal-type homeobox transcription factor 2 (CDX2). Still, it had a specificity that was significantly greater than that of CDX2 as an immunomarker of CRC, according to the findings of an immunohistological investigation that compared A33 with CDX2 (Davidsen, 2020).

#### 2.3.3 Cytokeratins (CKs)

CKs are proteins found in the cytoskeleton and located in intermediate filaments. It is a member of a group of approximately 20 cytoskeletal proteins frequently used as immune-histochemical markers in diagnosing CRC in tumors that have been generated from epithelia. In most cases, neoplastic cells maintain CK expression; specialized anti-CK antibodies are frequently used in histopathology diagnoses to trace tumor origins, especially in metastases (Hrudka et al., 2021). Cytokeratins, a group of intermediate filament proteins, have potential molecular mechanisms involving epithelial cell differentiation, tumor invasion, and metastasis, making them valuable biomarkers for cancer diagnosis and prognosis; currently, cytokeratin-based assays, such as CK19 for detecting disseminated tumor cells, are utilized in clinical practice for assessing the presence of minimal residual disease and predicting treatment response in various cancer types, including CRC. Two enzymes, CK7 and CK20, are frequently involved in CRC. Different types of glandular and ductal epithelia contain CK7. Simultaneously, CK20 is abundantly expressed in mucosal cells of the gastrointestinal and urinary tracts (Hrudka et al., 2022). Tissue expression of CK15 was significantly linked with CRC subtype and stage. A higher level of CK18 expression is found in CRC tumors compared to the normal colorectal tissue surrounding them (Sun et al., 2018). It is a single predictor of long-term survival in CRC patients when CK18 expression is upregulated in tumor tissue. The viability, migration, and invasion of CRC cells were decreased by downregulating CK18 expression (Jelski and Mroczko., 2020). All three of the detected cytokeratins 8, 18, and 19 can potentially be helpful biomarkers for the early diagnosis of CRC (Luo et al., 2020).

#### 2.3.4 Telomerase

A telomerase ribonucleoprotein increases the number of TTAGGG repeats at the ends of chromosomes to preserve telomere length. Intrinsic RNA serves as a scaffold for reverse transcription in telomerase. The telomere controls chromosomal stability and cell life span. The telomerase enzyme is present in human cancer cells (80%–90%) and differentiated cells, such as germ-line cells (Kibriya et al., 2022). Telomere length in cancer tissue was substantially shorter than in normal mucosa. Advanced CRC (stage II–IV) cancers have longer telomeres than stage I tumors (Ye et al., 2021). According to Taheri et al. (2022), CRC tissue had reduced hTERT expression levels. Since telomerase is present in healthy and malignant intestinal epithelial cells, measuring hTERT alone may underestimate its prevalence. Numerous studies have demonstrated that telomerase has a high-level telomere-specific reverse transcriptase (hTERT), which improves Nuclear factor erythroid 2-related factor 2 (NRF2) synthesis by recruiting Y-box binding protein 1 (YBX1) to trigger the NRF2 promoter, promoting CRC proliferation and migration. These findings provide a new conceptual underpinning for CRC treatment (Gong et al., 2021).

#### 2.3.5 Special AT-Rich sequence-binding protein 2 (SATB2)

SATB2 belongs to the group of transcription factors that bind to matrix attachment regions and control the development of the skeleton. The appendix and colon epithelium both had significant levels of SATB2 expression (Huang and Yang, 2022). SATB2, a transcription factor involved in gene regulation, has potential molecular mechanisms related to cell differentiation, cell migration, and tumor metastasis, suggesting its significance as a biomarker. However, SATB2 expression has been identified as a useful marker for CRC diagnosis and distinguishing primary colorectal tumors from metastatic tumors, and further research is needed to establish its full clinical utility and potential therapeutic implications. SABT2 has recently been identified as a biomarker for CRC, and various hereditary disorders connected to SABT2 have been reported (Mezheyeuski et al., 2020). SATB2 was positive in 83.7% of stage III/IV, 91.4% of stage II, and 92.4% of phase I colorectal adenocarcinomas, according to Dabir et al. (2018). According to multiple studies, when paired with conventional panels of CDX2, CK20, CK7, and cytokeratin 20, SATB2 is a highly specialized marker for CRC (Oh and Joo, 2020).

#### 2.3.6 Caudal type homeobox 2 (CDX2)

Intestinal epithelial cells express the homeobox protein CDX2 in their nuclei. As a trustworthy and accurate immunomarker for CRC, CDX2 is frequently used. It is believed that CDX2 is a tumor suppressor gene because it does not manifest itself in instances of CRC. By overexpressing CDX2 using an hTERT (hypoxia-inducible human telomerase reverse transcriptase) promoter-driven plasmid, colon cancer cells were prevented from progressing malignantly (Al-Duhaidahawi., 2023). The CDX2 gene promoter region’s methylation has been associated with a higher risk of CRC. The CDX2 gene promoter area was methylated in 78.5% of the CRC tissue. CDX2 downregulation was associated with high-grade, advanced cancers with liver metastases (Aasebø et al., 2020). Furthermore, disease-free and overall survival (OS) were considerably poorer in people with stage T4 CRC and low CDX2 expression (Choi et al., 2022).

#### 2.3.7 Methylation of DNA

At every stage of carcinogenesis, from polyps to colorectal adenocarcinomas, hypermethylation drives the transcriptional silence or downregulation of suppressor genes, which renders tumor suppressor genes inactive. In CRC, numerous genes, particularly those in the promoter region, including HLTF, CDH1, SEPT, VIM, TIMP3, CDK2A, SFRP2, SFRP1, MGMT, MLH1, and APC, are methylated (Mo et al., 2023). Suppression of histone deacetylation and demethylation are used in CRC cells to increase Syndecan-2 (SDC2) expression because the SDC2 promotor region is typically hypermethylated in CRC. Methylated SDC2 for the non-invasive diagnosis of CRC has reasonable specificity (88.2%–98%) and sensitivity (77.4%–90.2%) (Siri et al., 2022). The zinc finger protein 625, LON peptidase N terminal domain and ring finger 2, WD repeat domain 17, and syndecan 2 CpG island promoters were methylation in both cancer and laterally spreading tumor non-granular (LST NG). This indicates that the LST NG phase may be the first stage of colorectal carcinogenesis (Iwaizumi et al., 2023). N-Myc downstream-regulated gene 4 (NDRG4) influences cell development and differentiation. In CRC, the NDRG4 expression is downregulated (Cao et al., 2020).

### 2.4 Blood biomarkers

Biomarkers can be detected using immunohistochemistry or blood-based protein quantification techniques. Blood-based markers can serve as a convenient screening tool for CRC, as blood donation or collection is a relatively simple process.

#### 2.4.1 Circulating tumor cells (CTC)

CTCs are epithelial cancer cells in peripheral blood after they have spread from the primary tumor or metastases and entered the circulatory system. They can be utilized as biomarkers to detect CRC or as knowledge-dissemination pathways, enabling therapy decisions (Chan et al., 2023). In early-stage cancers, circulating CTCs vary from 1 to 10 cells/10 mL of blood and may be less. CTCs can be differentiated from normal blood cells by differences in size and shape. More research is needed to validate the findings. Necrosis releases circulating-free DNA (cfDNA) as significantly bigger fragments in tumor cells (Li et al., 2023). This resulted in promising findings when circulating cfDNAs were quantitatively examined using the ratio of longer to shorter DNA fragments or when the cfDNA integrity number was measured after CRC diagnosis. Although CTCs have been shown to have predictive value in CRC, their use in screening is controversial (Hendricks et al., 2021).

#### 2.4.2 Circulating tumor DNA (ctDNA)

ctDNA (circulating tumor DNA) and cfDNA have emerged as promising biomarkers in CRC. The presence of ctDNA and cfDNA in the bloodstream allows for non-invasive detection of specific genetic alterations, such as mutations and methylation patterns, providing valuable information about tumor burden, treatment response, and disease progression, thereby enabling personalized medicine approaches in the management of CRC patients (Lyu et al., 2022). ctDNA has received extensive evaluation as a promising indicator for liquid biopsy in detecting and assessing therapeutic responses for CRC. ctDNA released from necrotic or apoptotic tumor cells. Although normal nontumor cells also shed cfDNA into the bloodstream, the cfDNA from tumor cells (i.e., ctDNA) only accounts for less than 1% of total cfDNA in the blood (Peng et al., 2021). Tests that rely on the detection of circulating tumor DNA (ctDNA), also known as liquid biopsies, are susceptible to vulnerabilities. A study conducted by Min et al. (2023) in 2023 demonstrated that quantitative analysis of ctDNA and qualitative investigation of SEPT9 methylation effectively diagnose CRC. According to a study conducted by Perdyan et al. (2020) in 2020, ctDNA demonstrated an accuracy of 87.2% and a precision of 99.2% in identifying clinically significant KRAS gene mutations in a group of 206 patients with mCRC. Several studies have reported elevated levels of cfDNA in cancer patients. According to Raunkilde et al. (2022), most cfDNA fragments of 180–200 base pairs in length originate from tumor cells that have undergone necrosis or cell death (Raunkilde et al., 2022). Tumor-specific genomic changes, including microsatellite instability, loss of heterozygosity, methylation, and mutations, are present in cfDNA.

#### 2.4.3 Circulating MicroRNA (c-miRNA)

Small non-coding RNAs called microRNAs (miRNAs) control the expression of genes by attaching them to mRNA. C-miRNA has contributed to diagnosis. The dysregulation of miRNA activity causes a variety of diseases, including cancer. Diagnostic panels that combined single miRNAs as a CRC marker with combinations of those detected in serum or plasma miRNA indicators have been investigated recently (Abo-Elela et al., 2023). In comparison to serum, plasma has higher levels of miRNA. Hemolysis must be regulated in samples during the preanalytical stage of the experiment because it can change the amounts of circulating miRNA in samples by rupturing erythrocytes that transport miRNA. The only miRNAs that seem suitable as clinical markers are those with severe up- or downregulation (Liu et al., 2021). Nearly 2/3 of miRNAs were downregulated in CRC compared to normal mucosa. According to one study, the four-stage change from colorectal adenocarcinoma via high- and low-grade dysplasia in adenoma involved differential expression of 230 miRNAs (Sur et al., 2022).

#### 2.4.4 Long non-coding RNA (lncRNA)

Long non-coding RNAs (lncRNAs), made up of more than 200 nucleotides and cannot be translated into proteins, are involved in several biological processes, including differentiation, immunological responses, and chromosomal dynamics. Because lncRNAs can pass across cell membranes, they can be discovered in various bodily fluids, including blood, plasma/serum, and urine (He and Wu., 2023). Numerous lncRNAs are linked to the development of CRC and carcinogenesis at all stages. The WNT/-β catenin, PI3K/Akt, EGFR, NOTCH, mTOR, and TP53 signaling pathways are only a few carcinogenic signaling cascades that their changed expression can affect (Cai et al., 2019). The extracellular phospholipid-enclosed vesicles, known as apoptotic bodies, microvesicles, and exosomes, travel with lncRNAs in the blood. Exosomes, one of the three forms of extracellular vesicles, have the largest concentrations of long micro RNAs (lmiRNAs), which aid in tumor spreading, immunomodulation, and chemoresistance (Lulli et al., 2022). The first indicators discovered to have significantly higher expression in the plasma of CRC patients compared to healthy people were HOTAIR and CCAT1. Numerous more circulating lncRNAs, including NEAT1 variants 1 and 2, MEG3, PVT-1, 91H, Nbla12061, RP11-462C24.1, and LOC285194 have been identified as possible biomarkers for the identification of CRC (Zygulska and Pierzchalski., 2022).

#### 2.4.5 Pyruvate kinase muscle isozyme M2 (PKM2)

PKM2 is found in healthy and cancerous cells and is involved in energy metabolism. When PKM2 regulates the rate-limiting stage of glycolysis, tumor cells produce lactate rather than the normal respiratory chain for glucose metabolism (Cruz et al., 2021). There have been reports of PKM2 overexpression in colon adenomas, gastric cancer, and CRC. With its excellent sensitivity, PKM2 is adequate blood and fecal biomarker for CRC screening (Zahra et al., 2020). The study found that the fecal Tumor pyruvate kinase M2 isoform (tM2-PK) test had a 100% sensitivity and a 68% specificity in the tumor group. Specificity and sensitivity for the polyp group were 68% and 87%, respectively. The tM2-PK test is recommended as a non-invasive method to identify CRC and adenomatous polyps (Rigi et al., 2020).

#### 2.4.6 Dickkopf-related protein 3 (DDK3) and insulin-like growth factor binding protein 2 (IGFBP2)

DDK3, a tumor suppressor gene, and IGFBP2 a growth factor regulator, have potential molecular mechanisms involving cell cycle control, growth inhibition, and modulation of the IGF signaling pathway, highlighting their potential as biomarkers. However, further studies are needed to determine their clinical utility and application in cancer diagnosis, prognosis, and targeted therapies (Zygulska and Pierzchalski, 2022). The biomarker model can identify early-stage CRC with 95% specificity, 57% sensitivity for stage I, and 76% sensitivity for stage II. As a result, this panel of biomarkers recommends being used as a non-invasive blood screening and/or diagnostic test. It is comparable to a fecal occult blood test (FOBT) and FIT in CRC detection (Zygulska and Pierzchalski., 2022).

DDK3 (DNA-damage-inducible 3) and DDK1 (Dickkopf-1) are potential biomarkers in CRC. DDK3 expression has been associated with tumor suppressive effects, and its downregulation is often observed in CRC, suggesting its potential as a diagnostic or prognostic biomarker. On the other hand, DDK, an antagonist of the Wnt signaling pathway, is frequently overexpressed in CRC, and its elevated levels may serve as a biomarker for disease progression and therapeutic response in CRC patients (Akhlaghipour et al., 2021). The human Dickkopf family includes the proteins DDK-1, DDK-2, DDK-3, and DDK-4, and the specific protein Soggy (Sgy) related to DDK-3 is all TEMs (tumor endothelium markers). The tumor endothelium of CRC tissues displays more pronounced expression levels of the TEMs, a group of 46 genes (Safari et al., 2018). Wnt blocker genes are epigenetically inhibited in CRC, among several other factors. During cancer development, the Wnt signaling pathway is triggered. These antagonistic genes include DDK genes, which are hypermethylated in the promoter of CRC cells and epigenetically silenced (Kaur et al., 2012). According to one study, CRC that was DDK-3 positive had a considerably greater mean microvessel count (9.70 vessels) than cancer that was DDK-3 negative. Therefore, it is believed that DDK-3 is a pro-angiogenic mediator in the growth of neovascularization during the progression of CRC (Soheilifar et al., 2019).

IGFBP-2 is an extracellular protein that binds insulin growth factor 2 (IGF-2), which is involved in the development and spread of cancer through the action of heat shock protein 27. In patients with colon cancer, higher serum concentrations of IGFBP-2 are associated with neoplastic changes in the higher levels of carcinoembryonic antigen (CEA) and colon. Consequently, it has been suggested that monitoring patients with CRC includes measuring IGFBP-2 levels as a diagnostic indicator (Chen et al., 2021). Thus, by preventing cell division, IGFBP-2 overproduction during colorectal carcinogenesis slows the formation of tumors. The sensitivity may be improved by combining IGFBP-2 with additional biomarkers, such as CEA (Gligorijević et al., 2022). Zhu et al. (2019) found that IGFBP2 overexpression promoted CRC cell proliferation and migration by suppressing E-cadherin expression and enhancing cell growth. Additionally, more significant tumor sizes and lower OS rates were linked to higher plasma IGFBP-2 levels, demonstrating that IGFBP-2 may function as a prognostic and diagnostic biomarker for CRC.

#### 2.4.7 Septin 9 (SEPT9) gene methylation

The SEPT gene family in humans consists of 13 genes (SEPT1-SEPT13). SEPT9 methylation DNA is the most well-known blood biomarker. The molecular mechanism of SEPT9 gene methylation in CRC involves the aberrant addition of methyl groups to CpG islands within the gene’s promoter region. This hypermethylation leads to the silencing of the SEPT9 gene and subsequent loss of septin protein expression. The disrupted septin function contributes to defective cytokinesis, abnormal cell morphology, and increased genomic instability, promoting the development and progression of CRC (Baharudin et al., 2022). The detection rate for those with CRC stages 0-I using this method ranges from 57% to 64% (Zhao et al., 2020). According to the meta-analysis, individuals with advanced CRC cases were more likely to test positive for methylated SEPT 9 (mSEPT9) than those with early-stage CRC, and the opposite was true for people with early-stage CRC (Min et al., 2023). According to the latest meta-analysis released in 2020, the SEPT9 assay has a specificity of 92% and a sensitivity of 69% for CRC diagnoses (Hariharan and Jenkins, 2020).

### 2.5 Stool biomarkers

Stool samples are more suitable for the early detection of CRC than blood tests because exfoliating tumor cells appear in the large intestine or rectal lumen during colorectal carcinogenesis far earlier than the beginning of tumor cell vascular penetration.

#### 2.5.1 Stool DNA (sDNA)

The molecular mechanism of stool DNA methylation in CRC involves detecting aberrant DNA methylation patterns in the stool samples of patients. Abnormal methylation of specific genes associated with CRC can serve as a non-invasive biomarker for the early detection and screening of the disease (Mueller and Győrffy., 2022). The human genome makes up less than 0.01% of the total DNA in stools; the other 99.99% comes from gut bacteria or food. The DNA of tumor cells expelled with feces contains abnormal genetic and epigenetic changes, which may serve as biomarkers for the detection of cancer (Gao et al., 2023). Several genes such as WIF1, VIM, TFPI2, SFRP2, RASSF2A, NDRG4, MGMT, MLH1, MINT31, MINT1, KRAS, ITGA4, IRF8, ID4, HLTF, GSTP1, GATA4, ESR1, CXCL21, CRBP1, CDH13, CDKN2A, CDH1, BMP3, ATM, and APC have all been studied for CRC diagnosis (Park et al., 2017). There is a Multitarget stool DNA (mt-sDNA) test (Cologuard, which combines hemoglobin, NDRG4, KRAS mutations, and BMP3 DNA methylation) and a plasma SEPT9 DNA methylation test (Epi proColon) that has been utilized more extensively in clinical settings. In asymptomatic people, mt-sDNA testing has a sensitivity of 90% for detecting CRC. DNA testing has a specificity range of 86.6%–98% (Ladabaum et al., 2020). A colonoscopy is a next stage in diagnosing a colorectal tumor in the event of a positive mtsDNA test. Asymptomatic participants in an intriguing study endured CT colonography and an mt-sDNA test (FDA-approved). Overall, CT colonography screening had a considerably higher detection rate for advanced neoplastic lesions (5%) than the mt-sDNA test (2.7%). There were 0.31% and 0.23% overall detection rates for CRC (Pickhardt et al., 2020).

#### 2.5.2 Faecal immunochemical test (FIT)

The FOBT is modified into the FIT, which checks for blood that digestive proteolytic enzymes have broken down. Even though early CRC detection is crucial for reducing CRC mortality, there is limited data to support the stage-specific sensitivity of the FIT in CRC detection. The FIT detected stage I cancers with a sensitivity of 68% (95% CI, 57%–78%), stage II cancers with a sensitivity of 92% (95% CI, 87%–96%), stage III cancers with a sensitivity of 82% (95% CI, 73%–89%), and stage IV cancers with a sensitivity of 89% (95% CI, 80%–95%) (Niedermaier et al., 2020). FIT offers an extensive spectrum of susceptibility for all stages of CRC, ranging from 25% to 79% (Van Doorn et al., 2015). T3 sensitivity was 83%, and T1 sensitivity was 40% in those with greater severity of CRC (Hijos-Mallada et al., 2021).

#### 2.5.3 FIT and stool DNA test

The diagnostic tools, such as RNA- or DNA-based testing, studied in a community-based population were found to increase the efficacy of the FIT procedure. Another study found that mt-sDNA testing was more effective than FIT at identifying advanced adenomas and sessile serrated polyps (Tanriver and Kocagoncu., 2023). However, mt-sDNA had a lower overall specificity for detecting all lesions than FIT. According to reports, a DNA-FIT test boosted detection sensitivity for CRC to 97.5% and advanced adenomas to 53.1% (Xu et al., 2022).

#### 2.5.4 Stool miRNA

In CRC, ncRNAs are abnormally produced, and based on the genes or pathways they control downstream, they may act as oncogenes (oncomiRs) or tumor suppressors (tsmiRs). A novel therapeutic approach and testing biomarkers have been developed due to the possible discrepancy among miRNA profiles of CRC and the normal intestinal mucosa (Fonseca et al., 2021). The study of stool miRNA has some drawbacks. First, it can be challenging to standardize procedures due to daily changes in fecal characteristics (density, volume). Second, it is essential to distinguish between the three types of feces miRNAs: fecal colonocyte miRNAs, exosomal miRNAs from fecal exosomes, and cell-free miRNAs from fecal homogenates (Ahmed et al., 2019). In the feces of CRC patients, the miR-145 and miR-143 were downregulated, while miR135, the miR17-92 cluster, miR-106a, miR-92a, miR144, and miR-21 were upregulated (Sattar et al., 2022).

### 2.6 Urine-based biomarkers

Urine biomarkers can be obtained non-invasively without forcing the patients to attend the clinic. Urine containing various components is considered the most effective and ideal sample for medical examination. Additionally, because the urinary tract is highly clean physiologically, substances found in urine may be less contaminated by germs than those in feces. Metabolomics has been routinely employed to identify metabolic abnormalities in CRC patients’ tissue, serum, and urine materials. A recent metabolomic study discovered that CRC patients have a unique metabolic phenotype characterized by dysregulated expression of metabolites in glycolysis, the tricarboxylic acid (TCA) cycle, the urea cycle, tryptophan, arginine, proline, pyrimidine, polyamine, lactate, fatty acid, and amino acid metabolism, as well as gut microbial metabolism (Zhu et al., 2022; Qiu et al., 2023). Therefore, identifying urine biomarkers is desirable for diagnosing various cancers, including CRC (Iwasaki et al., 2019). ProstaglandinE2 and MicroRNA have demonstrated value in CRC detection. According to the available data, VOCs may be a possible biomarker for identifying CRC (Chandrapalan and Arasaradnam, 2020). Recently, researchers identified urinary metabolite biomarkers N1, N12-diacetyl spermine, hippurate, p-hydroxy hippurate, and glutamate as the best metabolites to discriminate CRC patients via low-cost point-of-care (POC) screening test (Zhou et al., 2022).

## 3 Targeted therapies in CRC

Early detection of colorectal tumors allows for successful management with first-line therapies such as surgery, radiation, or traditional chemotherapy. The 5-year OS rate for patients is 88%–92%, while it drops to 58%–72% for patients with stage IIIC. Even though traditional chemotherapy has a remarkable influence on cancer treatment, it is nevertheless significantly hindered by its nonspecific toxicity toward rapidly dividing cells (Mou et al., 2021). By interacting with particular genes or proteins involved in cell growth or apoptosis resistance, highly targeted medications that aim to eradicate cancer cells have been made possible thanks to the amazing advancements in molecular oncology in this field. Highly effective cancer medicines are helpful for cancer treatment since they have improved tumor selectivity and have fewer adverse effects than traditional cancer treatments (Xie et al., 2020). As a result of a better understanding of the mechanisms involved in the evolution and proliferation of cancer cells, targeted therapies and medications with action focused on these pathways/features have been developed.

An earlier meta-analysis examined chemotherapy’s effectiveness and safety outcomes with bevacizumab, panitumumab, or cetuximab in mCRC. It demonstrated that bevacizumab was more effective in treating right-sided mCRC. In contrast, cetuximab was more successful in treating left-sided RAS wild-type (WT) mCRC (Cai et al., 2022). Both cetuximab and panitumumab, two different monoclonal antibodies (mAbs) that target the epidermal growth factor receptor (EGFR), are frequently used either alone or in conjunction with chemotherapy to treat people with mCRC that has the RAS wild-type. Despite being often regarded as interchangeable, the two antibodies possess distinctive molecular compositions and can function therapeutically significant in various ways (Shim, 2011). While there is less research on cetuximab or panitumumab as first-line therapies for older patients with mCRC, these drugs may still be an option for those with the wild-type KRAS mutation. Cetuximab, either alone or in combination with irinotecan, had a benign toxicity profile in elderly patients with severely pretreated mCRC, and the efficacy was comparable to that reported in younger patients, according to two retrospective studies (Bouchahda et al., 2008; Fornaro et al., 2011). Another study suggests combining bevacizumab and capecitabine is a safe and effective treatment for elderly individuals with mCRC (Sastre et al., 2012). The preliminary results that are currently available show that patients with mCRC who receive cetuximab or panitumumab treatment had higher response rates and longer PFS when KRAS mutations are absent (Kim, 2015).

## 4 Novel tyrosine kinase inhibitors (TKI)

Small-molecule, oral drugs that target particular tumor-causing proteins have been available to treat colorectal malignancies since the turn of the millennium. These proteins that cause tumors are known as tyrosine kinases, and over 90 are in the human genome. Based on their structure, activity, and localization, these 58 can be further separated into the two significant classes of receptor tyrosine kinases (RTKs) and non-receptor tyrosine kinases (NRTKs) (Natoli et al., 2010). RTKs and NRTKs have both been linked to the emergence of CRC. Drugs that target these proteins have several distinct benefits over conventional chemotherapy. Blocking these enzymes can help prevent cancer cell development because they may be overactive or abundant in cancer cells (Piawah and Venook., 2019).

### 4.1 Receptor tyrosine kinases (RTKs)

**FIGURE 1:**
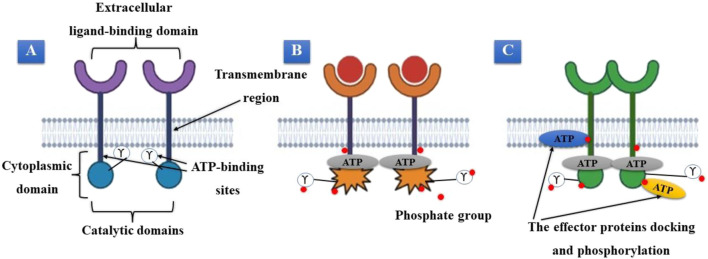
**(A)** Inactive tyrosine kinase receptor. **(B)** Activation of receptors, dimerization, and ATP binding. **(C)** The receptor’s phosphorylated tyrosines serve as a docking site for effector proteins.

The structural characteristics of the RTK superfamily of cell membrane proteins include an extracellular ligand-binding domain, a transmembrane region, and a cytoplasmic region, including ATP-binding and catalytic kinase domains. Based on similar receptor characteristics and/or shared ligands, at least 20 subfamilies of the approximately 60 RTKs have been discovered (Schlessinger, 2014). When peptide-based ligands transmit extracellular signals, these proteins are crucial for recognizing and transforming those signals. Their signals regulate cellular functions such as cell division, proliferation, and life span (Figure 1). RTKs function as monomeric transmembrane proteins when they are dormant. These proteins dimerize and create oligomeric pairs after becoming active. The receptors’ enzymatic domains must be activated to produce receptor oligomers, and their intracellular region’s tyrosine residues must be autophosphorylated (Figures 1A, B) (Diwanji et al., 2019). When ATP attaches to a specific receptor region, tyrosine residues on the receptor and effector proteins are phosphorylated. Many effector proteins involved in multiple signal transduction cascades associated with these receptors have been suggested to dock to the receptor’s phosphorylated tyrosines (Trenker and Jura., 2020). After docking, the receptor can activate these effector proteins via various phosphorylation processes. RTK activation requires the binding of ATP. If the ability of these receptors to bind and use ATP is impaired, their function will be significantly reduced. This is crucial for targeted therapeutic interventions (Figure 1C).

RTKs are crucial for controlling numerous cellular activities in a normal state. Still, when they express themselves abnormally, they can lead to uncontrollable cell division, contributing to cancer’s pathobiology (Wheeler and Yarden., 2014). Activating a particular subclass of RTKs subclass 1, or ERBB, is aberrant in epithelial cancers. Additional RTKs, including the tumor metastasis-promoting Platelet-derived growth factor (PDGF), VEGFR, and VEGFR2 receptors, appear essential for tumor growth (García-Aranda and Redondo, 2019).

### 4.2 Non-receptor tyrosine kinases (NRKTs)

The nucleus, the inner surface of the plasma membrane, and other cell components all include NRTKs, a sizable class of cytosolic proteins. By participating in cellular signaling cascades, these proteins play an essential role in controlling survival, migration, differentiation, cellular proliferation, and metabolism. Given the role of NRTKs in cells, it is unsurprising that the cell tightly regulates their activity (Siveen et al., 2018). When these proteins, like their receptor counterparts, fail to function correctly due to genetic mutation, resulting in overexpression, loss of autoregulatory processes, or abnormal signaling, they can lead to the pathophysiology of various cancer types. Therefore, it is unsurprising that this protein family has become a crucial therapeutic focus in the fight against cancer (Fox et al., 2019).

### 4.3 TKIs targeted in CRC

After the Food and Drug Administration (FDA) gave its approval for the TKI imatinib in 2001, there was a rise in people’s interest in protein kinase inhibitors (Huang et al., 2020). Imatinib’s anticancer activities have been confirmed by numerous *in vitro* studies against CRC, and in vivo experiments may validate these findings. Imatinib’s anticancer effects in CRC were synergistic and pleiotropic (Dhiman et al., 2020). The development of small kinase inhibitors is based on the structure and sequence of the kinase catalytic core, which is defined by the presence of a smaller N-terminal subdomain (N-lobe) made up of a long α-helix and a β-sheet. A big C-terminal subdomain (C-lobe) with a primarily α-helical structure and an ATP binding site that serves as a hinge during structural adjustments (Adnan et al., 2022). A highly conserved Asp-Phe-Gly (DFG) motif, a component of the ATP-binding site that regulates magnesium binding, follows the activation loop (A-loop), which controls kinase activity (Bhullar et al., 2018).

Kinase inhibitors are divided into two major categories based on how they work: In addition to competing for the primary ATP-binding domain of the kinase catalytic core in the active form, type-I and type-II small kinase inhibitors are designed to bind to an additional allosteric pocket close to the ATP-binding site in the inactive state. Even so, type-II inhibitors are more focused than type-I inhibitors (Zhao et al., 2021). Type-I and type-II inhibitors prevent the specific protein kinase from phosphorylating a substrate molecule and deactivating downstream signaling. Kinase inhibitors may inhibit unregulated cell growth or apoptosis inhibition because dysregulated kinases can lead to defective signaling that can cause uncontrolled cell growth and proliferation (Roskoski, 2015).

Tyrosine kinases influence cell growth, migration, differentiation, apoptosis, and death by phosphorylating certain amino acids on substrate enzymes. This alters the downstream signal transduction triggered by TKs Du and Lovly. (2018). Dysregulated signal pathways can result from mutations or other constitutive activation or inhibition processes, which can cause cancer. Therefore, blocking these early signals with TKIs can avoid abnormal behavior of mutant or dysfunctional TKs. In tandem with the development of targeted monoclonal antibodies, a greater understanding of the molecular underpinnings and oncogenic signaling of CRC growth has led to testing TKIs (Yang et al., 2022). These substances can inhibit key enzymes that regulate signaling pathways crucial for cell survival, proliferation, differentiation, and development. Several RTKs, or the pathways through which these kinases function, have prospective therapeutic targets that have been enhanced or altered in CRC. Numerous small molecule TKIs have been discovered and examined for their potential to treat CRC cancer (Jiao et al., 2018).

The US FDA has authorized more than 50 TKIs, though most of these TKIs exhibit encouraging results in CRC pre-clinical testing. In the clinic, most patients fail (Tauriello and Batlle., 2016). Various causes include the absence of complicated predictive pre-clinical models, a lack of understanding of the pharmacodynamics and pharmacokinetics of TKIs, and a lack of information on the tumor mutational background and heterogeneity, which can cause clinical failure. Despite being a primary contributing factor in CRC metastasis and a therapeutic target, the TME remains unclear, contributing to the discrepancy between pre-clinical and clinical results (Tolba, 2020).

### 4.4 TKIs as monotherapy

The 14 TKIs that had passed pre-clinical monotherapy testing were also investigated in a clinical trial for mCRC. Four TKIs (Lenvatinib, cediranib, cabozantinib, and apatinib) were identified as promising in non-randomized phase I/II studies, and two (fruquintinib and regorafenib) indicated therapeutic value in a randomized phase III study. 13 of them shown noticeable anti-cancer benefits in a pre-clinical setting (Han et al., 2020). Regorafenib is an oral multikinase inhibitor, that is, now approved for use in third-line mCRC therapy. It prevents tumor angiogenesis, oncogenesis, metastasis, and immunology by inhibiting tyrosine kinase receptors (Calvo-Garcia et al., 2022). Apatinib, an s-SRC, c-Kit, and VEGFR2, a relatively specific inhibitor, had a robust pro-apoptotic effect *in vitro* in animal CRC cell lines and humans. Patients with refractory CRC who did not have liver metastases responded well to apatinib alone, with a PFS of 3.9 months (Srivastava et al., 2022). A selective VEGFR1,-2,-3 inhibitors called fruquintinib has received approval in China to treat people with mCRC who have already failed at least two courses of systemic anti-neoplastic therapy. It is expected to be the 2nd TKI authorized for mCRC after receiving FDA fast-track approval for mCRC patients (Zhang et al., 2019).

Cediranib and cabozantinib, two TKIs that inhibit several kinases, produced apoptosis and antiproliferative activity in culture and slowed the growth of tumors *in vivo*. For CRC patients, it was not given any empirical investigation (Qin et al., 2019). Bosutinib is successful *in vitro* and *in vivo* in one analysis; however, its efficacy was only moderate in a following phase I clinical experiment (Isakoff et al., 2014). Numerous other TKIs also demonstrated encouraging anti-tumor pre-clinical effects, such as vandetanib (patient-derived cells), gefitinib (CRC cell lines), dasatinib (CRC cell lines and xenograft models), erlotinib (patient-derived xenografts), and linifanib (CRC cell lines, 3D micro tumor). However, none of these treatments worked effectively in the clinic as monotherapy (Table 1) (Lyer et al., 2022).

**TABLE 1:** The clinical results and pre-clinical efficacy of specific TKIs in monotherapy. The TKIs shown in bold have either demonstrated promising outcomes or have been effectively adapted for use in the clinic.

TKI	Target(s)	Study model	Clinical trial study	Clinical outcomes	Reference
vandetanib	VEGFR and EGFR families, TIE2, BRK, RET, and EPH receptor and Src kinase members	CRC Cell lines PDCs	open-label, randomized phase I trials	No OR observed	Kim et al. (2018)
sunitinib	RET, CSF-1R, FLT3, KIT, VEGFR1,2, 3, and PDGFRα and β	Cells with CRC Mouse Xenograft Model	A two-stage, multicentre, open-label study (Phase II)	No OR observed	Lu et al., 2021
regorafenib	Abl, PTK5, SAPK2, BRAFV600E, BRAF, RAF-1, Eph2A, Trk2A, DDR2, TIE2, FGFR2, FGFR1, PDGFRα and β, KIT, VEGFR1, 2, 3, RET	CRC PDTOs	Randomized, placebo-controlled, phase III analysis	The median OS for placebo vs. regorafenib was 6.4 vs. 5.0 months	Vlachogiannis et al. (2018)
nintedanib	FLT-3, CSF1R, VEGFR 1,2,3, FGFR 1–3, PDGFR α and β	cell lines of CRC	Randomized, double-blinded, placebo-controlled phase III experiment	Both of the study’s co-primary outcomes were not met. OS has not improved. PFS has significantly but modestly improved	Cheng et al. (2021)
linifanib	VEGF, PDGF, FLT3	CRC cell lines 3D micro- Tumors	An open-label, non-randomized study (Phase II)	No tumor responses were seen, and the ORR’s primary goal was not fulfilled	Chan et al. (2017)
lenvatinib	RET, KIT, FGFR, PDGFRα, VEGFR1, 2, and 3	PDX cell lines of CRC	single-centre, single-arm, phase II open-label trial	±PFS –3.6 months and ±OS—7.4 months, DCR (70.0%)	Iwasa et al. (2020)
gefitinib	IGF and PDGF-mediated signaling, EGFR exon 21 point mutation L858R, and exon 19 deletion	CRC cell lines	Phase II Randomized Trial	Median PFS is 1.9 months, with PR occurring in 1 of 110 patients (maximum 2.3 months)	Georgious et al. (2020)
fruquintinib	VEGFR1,2,3	Mouse model	a multicentre clinical trial, placebo-controlled, double-blind, Phase III randomized cohort	Fruquintinib *versus* placebo: median OS, 9.3 *vs*. 6.6 months Fruquintinib *versus* placebo: Median PFS, 3.7 vs. 1.8 months	Wang et al. (2020)
erlotinib	EGFR	PDX	Phase II study	No CR or PR	Rivera et al. (2021)
dasatinib	SRC family (FYN, YES, LCK, SRC) BCR-ABL, PDGFRβ, EPHA2, c-KIT	CRC xenograft Mice model CRC cell lines	Phase II multicentre trial	No OR observed	Scott et al. (2017)
cediranib	FGFRs, PDGFRs, VEGFR1, 2, 3	CRC cell lines Mouse model	Phase I, multicentre, open-label	DCR (81%)—26/32 patients	Melsens et al. (2017)
cabozantinib	TIE-2, FLT-3, TRKB, KIT, MER, TYRO3, ROS1, RET, AXL, VEGFR1, 2 and 3, MET	Models using Xenograft mice and CRC cell lines	Single-arm, two-stage Phase II trial	12-week PFS (34% of patients) 1 PR patient (Best response) SD with a DCR (72.7%) at week 6	Yang et al. (2022)
bosutinib	Hck, Lyn, Src, BCR-ABL	Models using Xenograft mice and CRC cell lines	Phase I, prospective clinical trial	CR—0 PR—1 ORR (6%)	Daud et al. (2012)
apatinib	s-SRC, c-Kit, VEGFR2	CRC cell lines Murine CRC cell lines	open-label, single-arm Phase II experiment	Average OS—7.9 months	Cai et al. (2020)
				Average PFS—3.9 months	

*PDX, patient-derived xenograft; PFS, progression-free survival; OS, overall survival; PDCs, patient-derived cells; PDTOs, patient-derived tumor organoids; DCR, disease control rate; ORR, objective response rate; OR, overall response; CR, complete response; SD, stable disease; PR, partial response; mCRC, metastatic colorectal cancer; CRC, colorectal cancer.

### 4.5 Utilization of TKIs in combination therapies

TKIs have been explored in CRC treatment combinations, and pre-clinical studies with 17 TKIs have been successful. Compared to the related pre-clinical investigations, several clinical trials employed various chemotherapy agents or antibodies with similar sites (Chen H. et al., 2022a; Chen R. et al., 2022b).

#### 4.5.1 TKIs combination with antibodies target

Targeting the EGFR/mitogen-activated protein kinase (MAPK) pathway, TKIs are combined with antibodies. In a previous study, cabozantinib was combined with the anti-EGFR antibody cetuximab to treat CRC cell lines, and it was found that this combination helped overcome cetuximab tolerance (Strickler et al., 2021). Furthermore, when TKIs are combined with an anti-VEGF antibody, such as bevacizumab or imatinib, a greater degree of vascular normalization has been observed without activation of extracellular matrix (ECM) deposition (Schiffmann et al., 2017).

#### 4.5.2 TKIs combination with immunotherapy

When CT-26 isografts were treated with lenvatinib, pembrolizumab, and an anti-PD-1 antibody, tumor growth *in vivo* was significantly inhibited. Regorafenib and nivolumab had a synergistic immune-modulatory effect on CRC cells in another instance (Kato et al., 2019). Anti-PD-1 antibody nivolumab was combined with regorafenib to promote anti-tumor activity (Doleschel et al., 2021). Combinations of regorafenib and ICI were the focus of subsequent research. However, the findings revealed no therapeutic value. It evaluated the potential effectiveness of the combination of pembrolizumab and lenvatinib in patients with advanced non-MSI-H mCRC (Wang et al., 2020).

#### 4.5.3 TKIs combination with radiotherapy

Encouraging results have been reported for the pre-clinical trials of cediranib in combination with radiation in CRC. In addition, vandetanib, irinotecan, and radiation significantly diminished tumor size in human colorectal xenograft models (Meyerhardt et al., 2012). However, the combination of vandetanib with cetuximab and irinotecan did not demonstrate any improvement in effectiveness compared to earlier results in patients with mCRC who had undergone therapy (Table 2) (Wachsberger et al., 2009).

**TABLE 2:** Clinical results of additional TKI selected from combination therapies in pre-clinical studies.

TKI	Clinical findings	Model of the trail	Target (s)	Clinical combination	Clinical trial study	Ref
vatalanib	ORR, OS, and PFS were not enhanced	CRC cell lines	VEGFR1,2, PDGFR, c- Kit, C-Fms	vatalanib + FOLFOX4	Phase III randomized, placebo-controlled	To et al. (2015)
vandetanib	PD (34%), SD (59%), PR (7%)	Xenograft Model	Families VEGFR and EGFR, TIE2, BRK, RET, and Src kinase family members and EPH receptor	vandetanib + irinotecan + cetuximab	Trial I comprising a larger MTD population	Eng et al. (2019)
	PFS average—3.6 months					
	Average OS—10.5 months					
sunitinib	PR—8/17 patients	Mouse model and CRC cell lines	RET, CSF-1R, FLT3, KIT, β, VEGFR1,2,3, and PDGFRα	sunitinib + FOLFIRI	Phase II Multicenter, open-label	Di Desidero et al. (2019)
sorafenib	No OR detected, 1.84 months for the median PFS	CRC cell lines	PDGFR-ß, VEGFR1,2,3, RET, RET/PTC, FLT- 3, c-CRAF, KIT, mBRAF, BRAF	sorafenib + cetuximab	open-label, single-arm Phase II trial	Rizzo et al. (2021)
semaxinib	PR confirmed PR (27%) and PR unconfirmed PR (18%)	murine model using xenografts and CRC cell lines	VEGFR2, c-kit	bolus 5-FU, leucovorin, and irinotecan (IFL) + semaxinib	Phase I/II trial	Woo and Jung. (2017)
pazopanib	FOLFOX6RR (38%) pazopanib + CapeOx + RR (40%) pazopanib	PDX Mouse Model	c-Fms, Lck, ltk, interleukin-2, FGFR 1 and 3, VEGFR1,2,3and KIT	FOLFOX6 or CapeOx + pazopanib	2-part, Open-label, dose-finding Phase I	Zhu et al. (2019)
nintedanib	Primary endpoint criteria were not met	CRC Cell lines & mouse model	FLT-3, CSF1R, VEGFR 1,2,3, FGFR 1–3, PDGFR α and β	nintedanib + mFOLFOX6	Phase II randomized, placebo-controlled	Boland et al. (2018)
gefitinib	OS, PFS, and OR did not improve than the FOLFIRI arm	CRC cell lines	IGF and PDGF-mediated signaling are mediated by EGFR exon 19 deletion or exon 21 point mutation L858R	gefitinib + FOLFIRI	A multicenter randomized trial (Phase II)	Palumbo et al. (2014)
erlotinib	Patients with BRAF and KRAS mutations did not respond. In KRAS/BRAF wt tumors, the RR was 52%, while in KRAS wt tumors, it was 41%	CRC cell lines	EGFR	erlotinib + cetuximab	Phase II trial	Xu et al. (2020)
dasatinib	Patients with high srcact expression had an ORR of 75%, while those with low srcact expression had a 0% rate	CRC cell lines	PDGFRβ, EPHA2, c-KIT, BCR-ABL, SRC family (FYN, YES, LCK, SRC)	dasatinib, capecitabine, oxaliplatin, and bevacizumab	dosage escalation in Phase I and cohort	Mettu et al. (2019)
cediranib	CR (41%), ECPR (53%)	Xenograft mouse model	FGFRs, PDGFRs, VEGFR1, 2, 3	cediranib + chemoradiotherapy	Phase I, alternating cohort design	Melsons et al. (2017)
bosutinib	SD or PR > 24 weeks (13% PR or SD) (all tumor types)	cell lines for CRC mouse xenograft models	Hck, Lyn, Src, BCR-ABL	capecitabine + bosutinib	open-label, dose-escalation, multicenter Stage I trial	Wenzel et al. (2020)
apatinib	DCR (22.2%), ORR (0%)	CRC cell lines Murine CRC cell lines	s-SRC, c-Kit, VEGFR2	apatinib + anti-PD-1 antibody SHR-1210	open-label, single-arm, Phase II prospective trial	Cai et al. (2020)
	±OS—is 7.80 months					
	±PFS—is 1.83 months					

*TFTD/TAS102, trifluridine/tipiracil; CapeOx/XELOX, capecitabine, oxaliplatin; 5-FU, 5-Fluorouracil; SN-38, the active metabolite of irinotecan; FOLFOX4/6/mFOLFOX6, leucovorin calcium (folinic acid), fluorouracil, and oxaliplatin; ECPR, excellent clinical or pathological response; PR, partial response/partial remission; MTD, maximum tolerated dose.

#### 4.5.4 TKIs combination with chemotherapy

5-fluorouracil (5-FU) treatment has enhanced survival in several cancers. The drug’s most significant effect has been documented in CRC. Active metabolites of 5-FU interfere with DNA and RNA synthesis via the folate metabolic pathway (Pardini et al., 2011). Patients with mCRC treated with oxaliplatin as a single drug showed limited efficacy, with response rates (RR) ranging from 10% to 24%. In contrast, the combination of oxaliplatin with 5FU has demonstrated RRs that vary from 20% to more than 50% due to a synergistic effect with 5FU (Comella et al., 2009). A camptothecin derivative known as irinotecan hydrochloride has anticancer efficacy against several tumor types. Irinotecan’s active metabolite is SN-38, which is synthesized by the enzyme carboxylesterase in the body. Survival has significantly increased since introducing irinotecan for treating CRC around the beginning of the 20th century. The overall survival time has been extended to more than 30 months due to the combination of irinotecan with 5-fluorouracil, oxaliplatin, and numerous molecularly targeted anticancer medications (Fujita et al., 2015).

The synthetic drug semaxanib, which inhibits VEGFR-1 and -2 tyrosine kinases, is a small and lipophilic molecule. For 28 patients with mCRC, semaxanib at two different dose levels in combination with fluorouracil and leucovorin showed a promising response of 31.6% as the first-line therapy (Rosen et al., 1999). A multicenter MABEL (Minimum anticipated biological effect level) trial studied the combination of cetuximab and CPT-11 at a dose and schedule as a pre-study in 1123 patients with mCRC exhibiting detectable EGFR. 9.2 months was the anticipated median survival, although at the expense of a tolerable toxicity profile. The efficacy and safety of C225 with CPT-11 observed in earlier studies were validated by MABEL in a larger context (Wilke et al., 2006). Gefitinib (ZD 1839) inhibits the EGFR tyrosine kinase selectively and has a 100-fold more effective potency against EGFR than other tyrosine or serine/threonine kinases. Gefitinib, unlike cetuximab, does not cause EGFR internalization or destruction in CRC cells, nor does it decrease EGF binding sites or EGFR protein levels (MacKenzie et al., 2005). Gefitinib monotherapy has shown anticancer activity in various CRC cell lines in both *in vitro* and *in vivo* investigations. Gefitinib, on the other hand, showed little activity in phase I/II clinical trials in individuals with mCRC (Rothenberg et al., 2004).

The most developed monoclonal antibody against EGFR currently under clinical development is cetuximab. A phase II trial of cetuximab with irinotecan was conducted in patients with EGFR-positive colorectal cancer who were refractory to both 5-fluorouracil (5-FU) and Irinotecan because preclinical and early clinical research indicate that cetuximab might reverse irinotecan resistance in CRC both *in vitro* and *in vivo*. The overall response rate for the 120 patients who received this regimen was 22.5% (Saltz et al., 2001). Kuo et al. reported results from a phase II research that included one cycle of FOLFOX-4, followed by further cycles of FOLFOX-4 with 500 mg/d gefitinib in 27 patients with proven progressing CRC following at least one chemotherapy regimen (generally irinotecan-based). 33% of patients experienced objective responses, whereas 48% maintained stable conditions over a prolonged period. The number of prior regimens exhibited no effect on response rates. The median event-free survival period was 5.4 months, and the total survival period was 12 months (Kuo et al., 2005).

## 5 Conclusion and future perspectives

CRC is a common cancer that substantially increases cancer mortality rates. Due to the complexity of colorectal carcinogenesis, the CRC survival rates of individual patients vary. It is thus beneficial to determine accurate and useful molecular biomarkers that contribute to CRC detection and management. Current studies have focused on finding precise, specialized biomarkers for CRC diagnosis and treatment success. This review article offered an overview of the latest CRC diagnostic biomarkers. Multiple signalling pathways are activated in CRC, making it impossible to address the disease with a single therapy. Combining conventional treatments with novel inhibitors that target multiple pathways is essential. Small-molecule TKIs are among the most recent additions to the vast range of cancer-treating drugs. TKI is a useful pharmacological strategy for treating a variety of malignancies, but it is not a solution. Protein tyrosine kinases appear to accelerate the growth and onset of CRC. The development of more effective biomarkers and the success of tailored medicines offer hope for the future management of CRC, but first, we need to learn more about the disease. Standardizing protocols, including extraction and quantification procedures and normalizing approaches, will be complicated in the future. Biomarker translation into the therapeutic context is also critical. Lastly, complicated laboratory equipment should be avoided for simple, inexpensive, quick solutions. We believed that developing additional novel targeted medicines could reduce the burden of CRC cancer.
